# Transcranial direct current stimulation (tDCS) applied to the left dorsolateral premotor cortex (dPMC) interferes with rhythm reproduction

**DOI:** 10.1038/s41598-017-11980-w

**Published:** 2017-09-14

**Authors:** B. Pollok, C. L. Overhagen, A. Keitel, V. Krause

**Affiliations:** Heinrich-Heine University Duesseldorf, Medical Faculty, Institute of Clinical Neuroscience and Medical Psychology, D-40225 Duesseldorf, Germany

## Abstract

Movement timing in the sub-second range engages a brain network comprising cortical and sub-cortical areas. The present study aims at investigating the functional significance of the left dorsolateral premotor cortex (dPMC) for precise movement timing as determined by sensorimotor synchronization and rhythm reproduction. To this end, 18 healthy volunteers performed an auditorily paced synchronization-continuation task with the right hand. A simple reaction time task served as control condition. Transcranial direct current stimulation (tDCS) was applied over the left dPMC in order to modulate cortical excitability either with anodal or cathodal polarity or as sham stimulation. TDCS was applied for 10 minutes, respectively on separate days. For the continuation task the analysis revealed significantly smaller inter-tap intervals (ITIs) following cathodal tDCS suggesting movement hastening as well as a trend towards larger ITIs following anodal stimulation suggesting movement slowing. No significant effect was found following sham stimulation. Neither for synchronization nor for reaction time tasks significant polarity-specific effects emerged. The data suggest the causal involvement of the dPMC in temporally precisereproduction of isochronous rhythms rather than sensorimotor synchronization. The present findings support the hypothesis that different cortical brain areas within the motor-control-network distinctively contribute to movement timing in the sub-second range.

## Introduction

Precise motor timing is crucial for a variety of everyday actions. While several movements like dancing are performed in synchrony with external cues, movement sequences can be precisely generated even in the absence of such cues. Brain imaging studies suggest that sensorimotor synchronization (i.e. movements with respect to external cues^1–3^; for reviews see refs 4–7) as well as sequence reproduction (i.e. movements with respect to internally generated cues^8–10^) engage a cerebello-thalamo-cortical network. The dorsolateral premotor cortex (dPMC) and mesial premotor areas are preferentially involved in the execution of repetitive tapping movements of the hands^11, 12^ as indicated by functional neuroimaging. While the dPMC has been particularly related to auditory-motor synchronization^1–3, 13^, for a review see ref. 14, evidence for the involvement in rhythm reproduction has been found as well^10, 13, 15^. Along these lines, the dPMC has been suggested to be part of a network that is engaged in sensory prediction, sensorimotor integration, and motor timing^16^. More precisely, it has been argued that a dPMC-parietal-cerebellar network is engaged in implicit timing that optimises movement execution with respect to predictable external events^17, 18^, for a review see ref. 19.

Increased activation of a certain brain area does not necessarily signify its causal contribution for task execution. Non-invasive brain stimulation techniques like transcranial magnetic and direct current stimulation (TMS/tDCS) allow an estimation of the functional significance of a certain brain area for a specific task by modulating cortical excitability (for a review see ref. 20). Anodal tDCS increases the likelihood of neural firing by depolarization of neurons resulting in increased excitability. Cathodal tDCS yields hyperpolarization of cell bodies resulting in reduced excitability (for reviews see refs 21–23). In a previous study, we used tDCS to investigate the causal involvement of the posterior parietal cortex (PPC) in the prediction of external events. TDCS applied to the superior part of the PPC corresponding to Brodmann area (BA) 7 was found to affect the accuracy of auditory-motor synchronization in a polarity-specific manner^24^. Interestingly enough, no significant effects on a simple reaction time task or on reproduction of the same isochronous sequence were found in that study^24^ suggesting the causal involvement of the PPC in auditory-motor synchronization rather than rhythm reproduction and basic motor control. This raises the question whether the dPMC – another key node within the brain network subserving precise motor timing – (*i*) is causally involved in and (*ii*) may distinctively contribute to task performance. We hypothesized that if (*i*) the dPMC distinctively contributes to movement timing in the sub-second range, tDCS should selectively affect synchronization or continuation performance. If (*ii*) the dPMC is on the other hand non-specifically involved in movement timing, tDCS may affect performance independent of the specific task.

## Results

Since stimulation conditions were correctly identified below chance level, we considered the blinding procedure to be successful. Kolmogorov-Smirnov tests suggest that in each condition the data were normally distributed (*p* > 0.10).

### Synchronization Task

Analysis of the tap-to-tone asynchrony revealed a significant main effect of *time* (*F*(1, 17) = 4.86, *p* = 0.04) suggesting larger asynchronies following tDCS (−72.28 ± 7.92 ms) as compared to pre-stimulation performance (−57.72 ± 7.13 ms). This effect was not modulated by tDCS polarity since neither *stimulation* (*F*(2, 34) = 0.19, *p* = 0.82) nor the *stimulation x time* interaction turned out to be significant (*F*(2, 34) = 1.53, *p* = 0.23).

**Figure 1:**
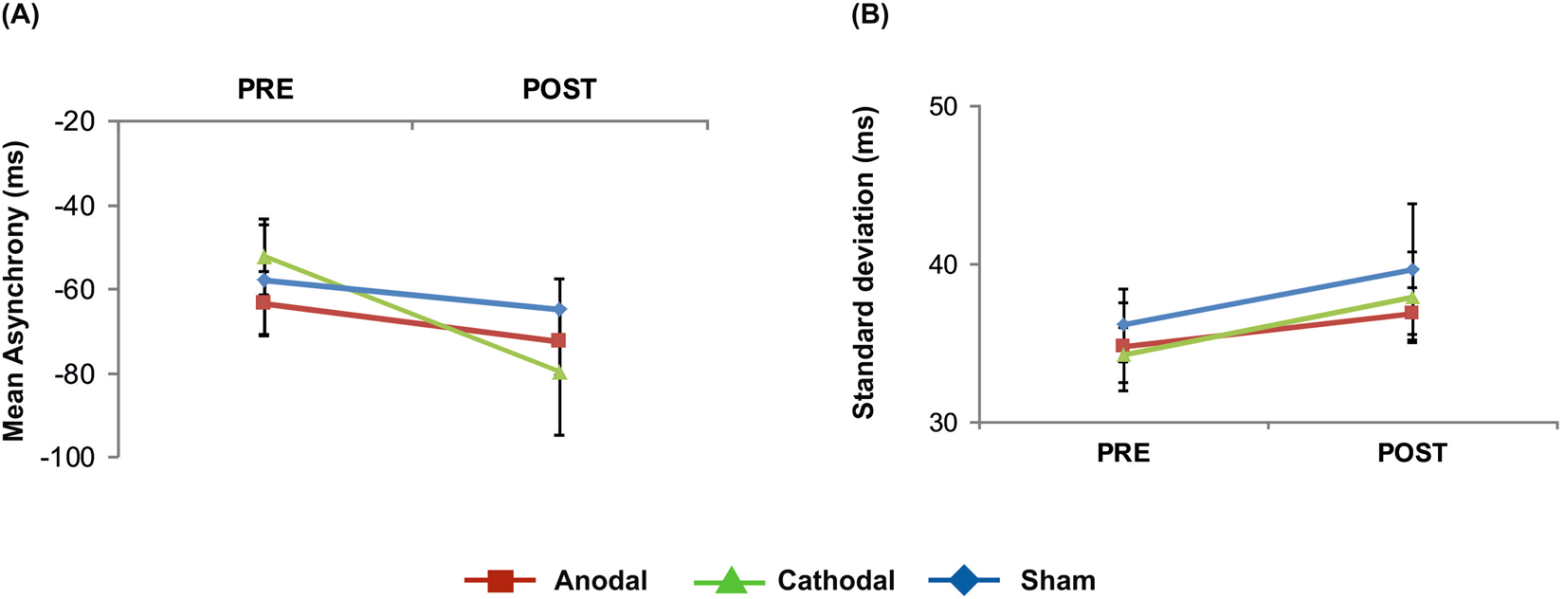
Effects of tDCS on synchronization accuracy: (**A**) Mean tap to tone asynchrony, (**B**) mean variability of the tap-to-tone asynchrony. Error bars indicate standard error of the mean (SEM).

Analysis of synchronization variability as determined by the standard deviation of tap-to-tone asynchronies revealed a trend towards significance of factor *time* (*F*(1, 17) = 3.91, *p* = 0.06), while neither *stimulation* (*F*(2, 34) = 0.45, *p* = 0.64) nor the *stimulation x time* interaction were found to be significant (*F*(2, 34) = 0.048, *p* = 0.91). The trend of factor *time* was due to slightly larger variability in post- (38.12 ± 1.61 ms) as compared to pre-tDCS measurements (35.02 ± 1.75 ms). Data are summarized in Fig. 1.

### Continuation Task

Analysis of mean inter-tap intervals (ITIs) revealed a significant *stimulation x time* interaction (*F*(2, 34) = 4.43, *p* = 0.02) while no significant main effects of *stimulation* (*F*(2, 34) = 0.65, *p* = 0.53) or *time* (*F*(1, 17) = 0.18, *p* = 0.68) emerged. Post-hoc analyses revealed significantly smaller ITIs following cathodal tDCS as compared to pre-stimulation performance (*t*(17) = 2.16, *p* = 0.046). Following anodal tDCS a trend towards larger ITIs was found (*t*(17) = −2.03, *p* = 0.058) while no significant pre-post difference following sham stimulation emerged (*t*(17) = 1.05, *p* = 0.31). Comparison of ITIs between tDCS conditions revealed significantly smaller values following cathodal as compared to anodal tDCS (*t*(17) = 2.16, *p* = 0.04) while no significant difference was found prior to stimulation (*t*(17) = −1.69, *p* = 0.11). Since ITIs prior to anodal tDCS were smaller as compared to both other tDCS conditions – at least on a descriptive level – we additionally calculated relative changes of post- with respect to pre-stimulation performance in order to account for these differences. The analysis of relative changes suggests a main effect of *stimulation* (*F*(2, 34) = 4.53, *p* = 0.02) indicating an ITI increase following anodal (*t*(17) = 2.10, *p* = 0.05) and a decrease following cathodal tDCS (*t*(17) = −2.10, *p* = 0.05) while no significant modulation in the sham condition was observed (*t*(17) = −0.94, *p* = 0.36). This analysis supports the findings found in the analysis of absolute values.

**Figure 2:**
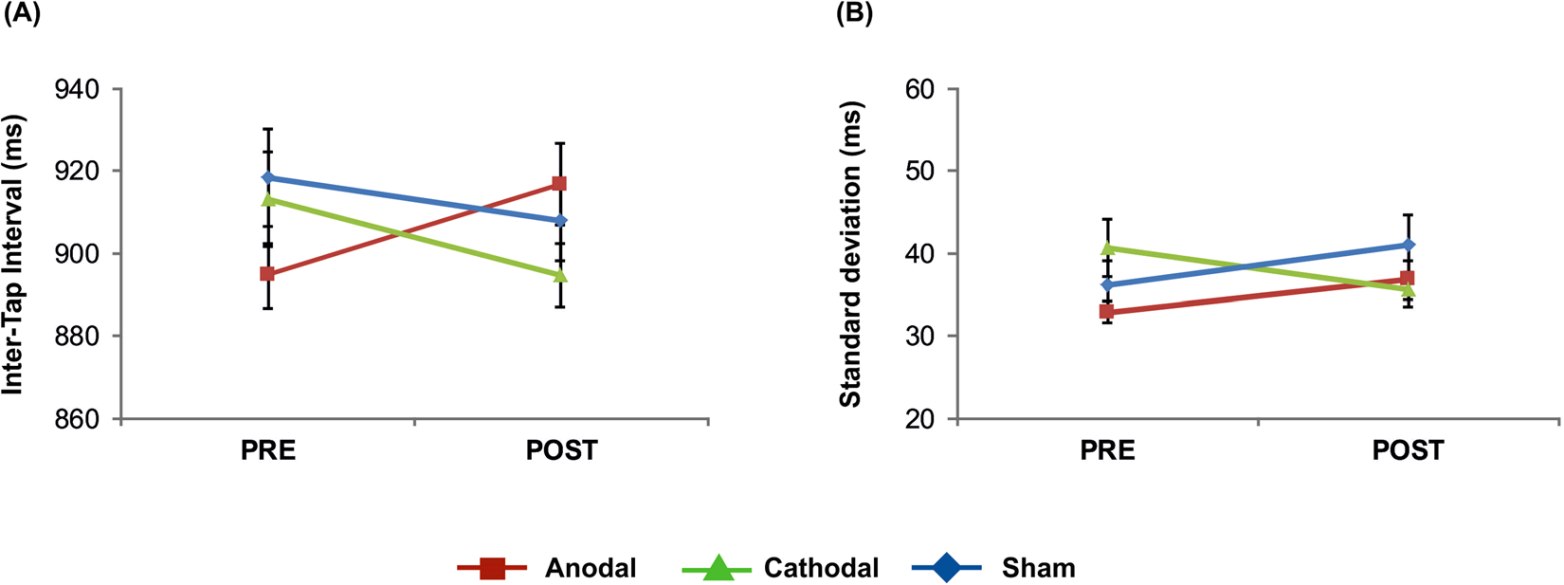
Effects of tDCS on continuation accuracy: (**A**) Mean inter-tap interval, (**B**) mean inter-tap interval variability. The analysis revealed significantly smaller ITIs following cathodal tDCS and a trend towards significantly larger ITIs following anodal tDCS. No significant modulation was found following sham tDCS. Error bars indicate standard error of the mean (SEM).

Analysis of ITI variability again showed a significant *stimulation x time* interaction (*F*(2, 34) = 3.78, *p* = 0.03) while neither *stimulation* (*F*(2, 34) = 2.07, *p* = 0.14) nor *time* (*F*(1, 17) = 0.42, *p* = 0.52) turned out to be significant. Post-hoc analyses suggest that the interaction was mainly driven by significantly larger ITI variability prior to cathodal as compared to anodal tDCS (*t*(17) = −2.92, *p* = 0.01). The remaining comparisons did not reach significance (*p* > 0.14). Results are summarized in Fig. 2.

### Reaction Time Task

The analysis of mean reaction times did neither reveal significant main effects (*stimulation*: (*F*(2, 34) = 0.97, *p* = 0.39); *time*: (*F*(1, 17) = 1.79, *p* = 0.20)) nor a significant *stimulation x time* interaction (*F*(2, 34) = 1.96, *p* = 0.16).

**Figure 3:**
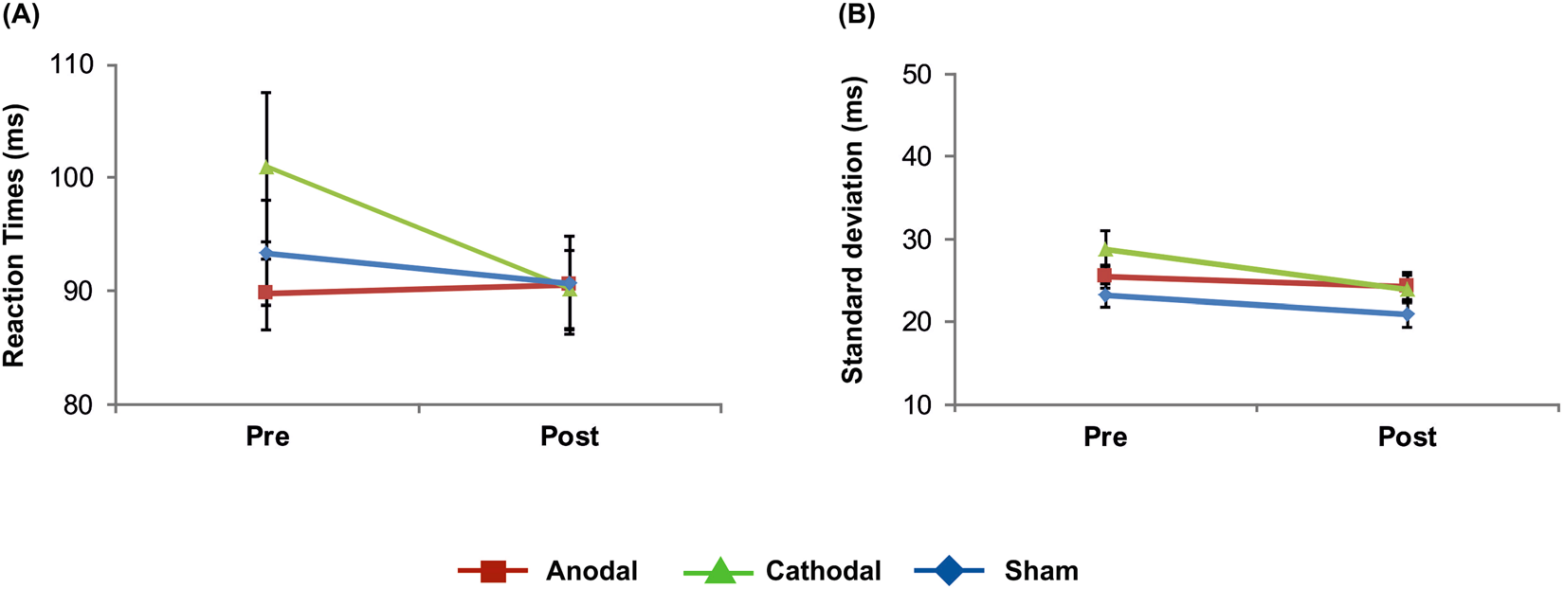
Effects of tDCS on reaction times: (**A**) mean reaction times, (**B**) mean reaction times variability. No significant polarity-specific effects were obtained. Error bars indicate standard error of the mean (SEM).

Analysis of reaction time variability revealed main effects of *stimulation* (*F*(2, 34) = 4.22, *p* = 0.02) and *time* (*F*(1, 17) = 7.90, *p* = 0.01), but not a significant *stimulation x time* interaction (*F*(2, 34) = 1.24, *p* = 0.30). The main effect of *stimulation* was due to significantly smaller ITI variability in the sham condition (22.05 ± 1.18 ms) as compared to anodal (24.81 ± 1.44 ms; *t*(17) = 2.21, *p* = 0.04) and cathodal tDCS (26.36 ± 1.68 ms); *t*(17) = 2.84, *p* = 0.01). No significant differences between anodal and cathodal tDCS emerged (*t*(17) = −0.90, *p* = 0.38). The main effect of *time* occurred due to smaller variability pre- (25.78 ± 1.78 ms) as compared to post-tDCS measures (23.03 ± 1.24 ms). Results are summarized in Fig. 3.

## Discussion

The present study aims at investigating the functional significance of the left dPMC for precise movement timing in the sub-second range as determined by synchronization, continuation, and reaction time tasks. We hypothesized that if (*i*) the dPMC distinctively contributes to movement timing in the sub-second range, tDCS should selectively affect synchronization or continuation performance. If (*ii*) the dPMC is unspecifically involved in movement execution, tDCS should affect performance independent of the specific task. The analysis revealed a significant polarity-specific modulation of continuation performance, while neither reaction times nor synchronization accuracy was significantly modulated by tDCS. Results from the present study signify the causal involvement of the left dPMC in rhythm reproduction of the right hand rather than auditory-motor synchronization and basic motor control.

The analysis of reaction times as well as synchronization performance did not reveal significant polarity-specific tDCS effects. The former finding is in line with results from previous TMS-studies^25–28^ suggesting that the dPMC controls movement timing in tasks with higher demands, but not in simple reaction time tasks. Regarding synchronization performance data from neuroimaging studies indicate the involvement of a cerebello-thalamo-cortical network in the synchronization of finger taps with respect to an external pacing signal^16^ (for reviews see refs 4–6). Although the same network seems to be involved in rhythm reproduction without external pacing^8–10^, rhythm reproduction has been rather related to a sub-network including the basal ganglia – in particular the putamen – the supplementary motor area (SMA) and the PMC^29^.

Chen and colleagues^3^ found evidence for the assumption that the dPMC might interface sensory – in particular auditory – cues with temporally organized movements. Those data suggest a specific significance of the dPMC for precise auditory-motor synchronization in particular with respect to complex rhythms. Studies investigating a possible contribution of the dPMC for auditory-motor synchronization with respect to isochronous rhythms revealed mixed results: While the majority of studies using functional magnetic resonance imaging (fMRI)^3, 30, 31^ and TMS^32, 33^ do not support the involvement of the dPMC in such tasks, two TMS-studies suggest that the dPMC subserves auditory-motor synchronization with respect to isochronous sequences – at least in the millisecond range^34, 35^. Although the present ANOVA did not support this hypothesis, larger tap-to-tone asynchronies following cathodal stimulation were found – at least on a descriptive level. The comparison between pre- and post-tDCS asynchrony in the cathodal condition revealed significantly larger values post-stimulation (*t*(17) = 2.81, *p* = 0.01) while no significant differences were found following anodal and sham tDCS (p > 0.33). We realize that this comparison is not justified by the results from the ANOVA, but this finding is – on principle – in line with our previous data^35^ and those from Bijsterbosch and colleagues^34^ using TMS. We therefore cannot definitely rule out the possibility that the dPMC is involved in auditory-motor synchronization, but the present results are in favour of a superior role for rhythm reproduction.

The present data indicate that tDCS selectively interferes with continuation performance suggesting the causal involvement of the dPMC in the reproduction of an isochronous rhythm. A previous study using positron emission tomography (PET) suggests the involvement of premotor areas for the execution of simple self-initiated finger movements^36^. This finding is in line with data from patients with lesions in premotor areas showing impaired reproduction of a rhythm from memory while synchronization with respect to an external pacing signal was unimpaired^37^. The patients with PMC lesions were most severely impaired when they had to reproduce rhythms with both hands in an alternating manner^37^. This finding implies that premotor areas might be particularly relevant for the execution of complex movements of the upper limbs. In line with this hypothesis a previous rTMS study suggests that the SMA is involved in interhemispheric communication and thereby modulates the interaction between both hands in bimanual tasks of increasing complexity^38^. In line with those observations, dorsolateral premotor areas as well as the pre-SMA have been shown to be associated with the selection of timing parameters while centro-medial and prefrontal areas might be stronger related to error monitoring and correction^39^.

Noteworthy, the SMA has been particularly related to the execution of self-initiated movements as shown in human^40, 41^ and animal^42^ studies. In contrast to this, lateral parts of premotor areas have been related to the execution of externally triggered movements^43^ while direct comparisons during self-initiated versus externally paced movements failed to support this functional dichotomy in humans^40, 44, 45^ and animals^46, 47^. The data from Cunnington *et al*.^40^ showed that although the magnitude of the hemodynamic response did not differ between tasks (self-initiated vs. externally paced movement), activation of the pre-SMA started around 1.5 s earlier in self-initiated than in externally paced movements supporting the hypothesis that the pre-SMA is crucially involved in early-stage preparatory processes of movements, presumably representing an internal control mechanism. Although this mechanism is essential for proper execution of self-initiated movements or movements with respect to external events that occur at relatively slow frequencies, it might not be adjuvant for rapidly initiated movements with respect to unpredictable external events in particular occurring at relatively fast rates. In such cases movement execution may rely little on internal control mechanisms as provided by the SMA. This interpretation is in line with results from a previous study investigating the neuronal underpinnings of timing and force control during precision grip^48^. The SMA was associated with the interaction of timing and force variation while the dPMC was found to process both aspects, separately, but not their integration. This finding implies that the SMA might be stronger related to higher level motor control while the dPMC seems to be associated with its lower level aspects. In line with this interpretation, the pre-SMA has been related to the selective initiation of movements (i.e. what to do) while the dPMC might account for movement execution^49^. Based on these findings, one might speculate that the proposed functional dichotomy between the SMA and the dPMC regarding self-initiated and externally paced movements might rather reflect differences in preparatory processes.

Interestingly enough, the present data suggest movement hastening – as indicated by smaller ITIs – following cathodal tDCS, while following anodal tDCS the reversed pattern emerged. Although unexpected, this finding agrees well with the observation that tDCS modulates excitability of cortical inhibitory, rather than excitatory circuits^50^.

The present data are at odds with a previous TMS study suggesting that particularly the right dPMC is crucial for auditory-motor synchronization as well as continuation of a given rhythm^33^. In that study the participants synchronized their finger taps with respect to isochronous and metrical rhythms of higher complexity and continued tapping at the requested rhythm. While repetitive TMS (rTMS) applied at 1 Hz over the right dPMC was found to disturb auditory-motor synchronization of more complex rhythms neither left nor right dPMC rTMS affected performance in a continuation task with respect to an isochronous rhythm. Although the reasons for this discrepancy are not entirely clear, it is likely that different ITIs (900 ms in the present study vs. 250 ms used by Giovanelli and co-workers^33^) may have contributed to the conflicting results. Accordingly, evidence for the assumption that brain processes vary depending on interval duration was provided in humans^51^ as well as in monkeys^52^.

Compiling the present data with those from our previous studies investigating the functional contribution of the PPC for precise movement timing^24, 53^, we conclude that within the cerebello-thalamo-cortical network underlying motor timing in the sub-second range, different brain areas distinctively contribute to task execution: While the left PPC seems to be particularly involved in auditory-motor synchronization of movements with respect to a regular pacing signal, findings from the present study support the hypothesis that the dPMC is stronger involved in the reproduction of isochronous rhythms. This implies that although auditory-motor synchronization and rhythm reproduction involve the same brain network, proper task execution requires the integrity of different key brain areas: While the PPC seems to be stronger related to the synchronization with respect to the occurrence of a regular external event, the dPMC seems to be stronger related to the reproduction of a given rhythm. In addition, results from these studies indicate that – despite its relatively low spatial accuracy – tDCS is suitable for the selective modulation of cortical excitability even of adjacent brain areas.

### Limitations

One limiting factor of the present study is set by the pre-stimulation difference of ITIs in the continuation task. Although statistics did not reveal evidence for significant differences between tDCS conditions, Fig. 2A suggests smaller ITIs prior to anodal tDCS as compared to cathodal and sham stimulation, a finding that contributes to the significant *time x stimulation* interaction. In order to account for this pre-stimulation difference, relative changes were additionally calculated and the results support the findings of the analysis of the absolute values. Nevertheless, we would like to stress that – although the exact mechanisms are not well-understood – baseline differences represent an important factor influencing the effects of non-invasive brain stimulation methods like tDCS. Another important limitation is that we did not stimulate the SMA as control site to allow a conclusive answer on the question for functional differences between the dPMC and the SMA for movement timing. Nevertheless, based on findings from previous studies, we would argue that the SMA might be stronger related to early-stage motor preparation relevant for the execution of self-initiated and complex movements, while the dPMC might be associated with lower-level motor control. In other words, the SMA might be involved in preparation while the dPMC is involved in programming of movements. In relatively easy tasks with additionally short ISIs like the one used in the present study, movement execution might not rely on internal control mechanisms as provided by the SMA. As a consequence, tDCS applied to the dPMC interferes with task execution while we would expect that the same stimulation applied to the SMA would not result in behavioural effects. We realize that this interpretation is highly speculative at the moment and a direct comparison of stimulation effects associated with SMA and dPMC tDCS is needed in order to prove a possible dichotomy of the functional contribution of both areas for precise motor timing. We here focused on the functional role of the dPMC in order to determine whether and to what extent this area is distinctively involved in motor timing, particularly in comparison to the PPC.

A further limitation can be seen in the fact that tDCS was applied to the left dPMC, only, neglecting a possible contribution of the ipsilateral dPMC for precise motor timing. We decided to focus on the left hemisphere due to our previous studies^35, 54^ suggesting a crucial role of the left but not the right dPMC for precise motor timing of either hand. Moreover, findings from Sadato and co-workers^55^ suggest that premotor areas of the right hemisphere are particularly involved in the production of longer sequences leading to the hypothesis that right dPMC activation might represent the storage of motor sequences in working memory^12^ which does not appear to be relevant in relatively simple synchronization-continuation tasks as used in the present study. But, we acknowledge that stimulation of the right dPMC would have supported this hypothesis. Finally, we would like to stress that the rhythm reproduction task used here was very easy. It is reasonable to assume that higher task complexities would have revealed more pronounced effects. But, in order to achieve a better comparison with our previous studies^24, 53^, we decided to use the same tasks. Despite these limitations, the present findings should be seen as first evidence for the hypothesis that within the brain network underlying motor timing different brain areas distinctively contribute to task performance.

## Conclusion

Findings from the present study suggest the causal involvement of the left dPMC in the temporally precise reproduction of isochronous rhythms supporting the hypothesis that different brain areas within the motor-control-network distinctively contribute to movement timing of the right hand in the sub-second range.

## Material and Methods

### Participants

Eighteen healthy volunteers (9 male) aged between 21 and 27 years (22.8 ± 0.4 years; mean ± standard error of the mean; SEM) participated in the study. Handedness was assessed by means of the Edinburgh Handedness Inventory^56^. The mean lateralization index was 98.9 ± 0.4 indicating that all participants were right-handed. They gave their written informed consent prior to participation. Volunteers with individual or family history of epileptic seizures or other severe neurological, psychiatric or internal diseases were excluded. The study was approved by the ethics committee of the medical faculty of the Heinrich-Heine University (study number 3347) and was conducted in accordance with guidelines set by the latest version of the Declaration of Helsinki.

The participants were blinded regarding the exact hypothesis of the study and the respective type of stimulation. Information was given after completion of the entire experiment.

### Paradigm

The participants performed a synchronization task followed by a continuation task as well as a simple reaction task. The synchronization task requires the subjects to tap in synchrony with an isochronous auditory pacing signal presented with a stimulus onset asynchrony (SOA) of 900 ms (100 ms length). After 30 taps the tone stopped and the participants were instructed proceeding tapping at the same interval for another 30 taps. The synchronization task was always followed by the continuation task. For the simple reaction task the same tone was presented with irregular SOAs of 1.000, 1.500, or 2.000 ms. The participants were instructed to react as fast as possible as soon as the tone appeared. The order of reaction time and synchronization-continuation tasks was counterbalanced across participants and tDCS conditions.

Behavioural data were measured by a photoelectric barrier mounted on a pad prior to and immediately after tDCS. E-Prime^®^ 2.0 software (Psychology Software Tools, Sharpsburg, USA) was applied for tone presentation and acquisition of behavioural data. In short practice runs prior to data acquisition the subjects were familiarized with the respective tasks. During the experiment the subjects were comfortably seated in a reclining chair and were instructed to keep their eyes open and to relax.

### Transcranial direct current stimulation (tDCS)

TDCS was applied through rubber electrodes nestled between saline-soaked sponges placed on the skin surface. The stimulation electrode (3 × 3 cm^2^) was placed above the left PMC and the reference electrode (7 × 5 cm^2^) above the right orbit. Self-adhesive bandages (Coban, 3 M Deutschland GmbH, Neuss, Germany) were used for fixation of the electrodes. TDCS was applied using a battery-driven DC-Stimulator (DC-Stimulator Plus, NeuroConn, Ilmenau, Germany) for 10 minutes plus fade-in and fade-out periods of 10 seconds, each. Intensity was set to 0.25 mA corresponding to a current density of 0.028 mA/cm^2^ below the stimulation electrode and 0.007 mA/cm^2^ below the orbito-frontal reference electrode. For sham stimulation, the stimulator automatically switched off after 30 seconds of either anodal or cathodal stimulation yielding sensations typically elicited by tDCS. Due to the short stimulation period, effects on neuronal excitability can be widely excluded. Impedance was kept below 10 kΩ (mean impedance: 9.74 ± 0.44 kΩ (sham), 9.71 ± 0.51 kΩ (anodal), 8.84 ± 0.49 kΩ (cathodal)). No significant differences between conditions were found (*F*(2, 34) = 2.06, *p* = 0.14). In order to exclude carry-over effects from the preceding tDCS, experimental sessions were separated by at least one week. Blinding of the main investigator was achieved by a second investigator who was responsible for running the DC stimulator only. The stimulator was covered by a paperboard in order to hide the stimulator.

To ensure that subjects were blind regarding the exact stimulation type, a stimulation questionnaire was completed after each session. Subjects were asked to estimate whether they had received active or sham stimulation. If active stimulation was chosen, they were asked to estimate whether anodal or cathodal stimulation was applied. Verum stimulation was correctly identified in 30%. Hit rate in the sham condition was 11%.

### Neuronavigation

The left dPMC was localized using a neuronavigation system (LOCALITE, Sankt Augustin, Germany). The target area was pre-defined by Talairach coordinates −29, 5, 47 (x, y, z) corresponding to Brodmann area 6.

To ensure that the target area for stimulation was sufficiently separated from the left M1, the cortical representation of the right first dorsal interosseus (FDI) muscle was localized using single pulse TMS. To this end, a standard figure of eight coil (MC-B70) connected to a MagPro stimulator (Mag Venture, Hückelhoven, Germany) was placed tangentially to the scalp to trigger motor-evoked potentials (MEP). The area evoking the largest motor response was defined as motor hot spot. In two sessions, M1 localization was not possible. The mean distance between M1 hot spot and the PMC target area was 5.11 ± 0.13 cm (sham), 5.08 ± 0.20 cm (anodal) and 4.89 ± 0.21 cm (cathodal). The distance did not differ significantly between sessions (*F*(2, 30) = 0.456, *p* = 0.638).

### Data analysis

Synchronization accuracy was determined by means of the tap-to-tone asynchrony and synchronization variability. The tap-to-tone asynchrony is defined as the mean temporal distance between tap and tone onsets. Synchronization variability is defined as the mean standard deviation of the asynchrony. Continuation accuracy was assessed by calculating the inter-tap interval as well as its standard deviation. In the reaction time task the temporal distance between tap and tone onsets was calculated as a measure of reaction times. Again, the standard deviation was calculated in order to estimate behavioural variability.

### Statistics

Data were analyzed by means of analyses of variance (ANOVA) with within-subject factors *stimulation* (anodal vs. cathodal vs. sham) and *time* (pre- vs. post-tDCS) using IBM SPSS 23. For post-hoc comparisons t-tests for dependant samples were adopted. Kolmogorov-Smirnov tests were implemented to control for Gaussian distribution. P-values were corrected for multiple comparisons using the sequential Bonferroni correction^57^.

### Data availability

The datasets generated during and/or analysed during the current study are available from the corresponding author on reasonable request.
